# Clinician and patient barriers to the recognition of insomnia in family practice: a narrative summary of reported literature analysed using the theoretical domains framework

**DOI:** 10.1186/s12875-019-1070-0

**Published:** 2020-01-04

**Authors:** Rowan P. Ogeil, Samantha P. Chakraborty, Alan C. Young, Dan I. Lubman

**Affiliations:** 1 Eastern Health Clinical School and Monash Addiction Research Centre, Monash University, Melbourne, Australia; 2 Turning Point, Eastern Health, 110 Church St, Richmond, Victoria 3121 Australia; 3 Department of General Practice, School of Primary and Allied Health Care, Monash University, Melbourne, Australia; 4 Eastern Clinical Research Unit, Monash University, and Eastern Health, Melbourne, Australia

**Keywords:** Insomnia, Family practice, Clinician-factors, Patient-factors, Theoretical domains framework

## Abstract

**Background:**

Insomnia is a common sleep complaint, with 10% of adults in the general population experiencing insomnia disorder, defined as lasting longer than three months in DSM-5. Up to 50% of patients attending family practice experience insomnia, however despite this, symptoms of insomnia are not often screened for, or discussed within this setting. We aimed to examine barriers to the assessment and diagnosis of insomnia in family practice from both the clinician and patient perspective.

**Methods:**

The present article identified research that has examined barriers to assessing insomnia from the clinician’s and the client’s perspectives following MEDLINE and Google Scholar searches, and then classified these barriers using the theoretical domains framework.

**Results:**

The most common barriers from the clinician’s perspective were related to Knowledge, Skills, and the Environmental Context. From the patient perspective, barriers identified included their Beliefs about the consequences of Insomnia, Social Influences, and Behavioural Regulation of Symptoms.

**Conclusions:**

Utilising this theoretical framework, we discuss options for bridging the gap between the identification and subsequent management of insomnia within the family practice setting. To assist clinicians and those in community health care to overcome the Knowledge and Skills barriers identified, this article provides existing relevant clinical criteria that can be utilised to make a valid diagnosis of insomnia.

## Background

Chronic insomnia (ICSD-3) or insomnia disorder (DSM-5) is characterised by poor sleep quality or inadequate sleep, lasting for > 3 months, accompanied by impaired daytime functioning [1, 2]. Insomnia is common, affecting up to 1 in 3 adults in the UK [3], North America [4], Europe [5]**,** and Australia [6], with an estimated 10% of the population suffering from chronic insomnia symptoms [7, 8]. Insomnia has negative effects on the health and quality of life of an individual [9]. Insomnia is also associated with a substantial economic burden to the community [10, 11], estimated to exceed $63 billion in the United States alone [12]. This burden includes not only health and direct medical-related costs, but costs due to lost productivity and/or errors attributable at work, or while driving that are attributable to sleep loss [13].

Insomnia is one of the most commonly encountered sleep disorders by clinicians in family practice (general practice in some jurisdictions) [14]. A recent study suggests that prevalence of insomnia complaints may be increasing, with up to 50% of patients who present to family practice suffering from insomnia [15]. Few patients presenting with insomnia to general practitioners (GPs) receive a referral for non-drug treatment, despite these treatments having the highest level of evidence for effectiveness [16]. Furthermore, the consequences of chronic insomnia are often not discussed by clinicians and patients in this setting [17–19].

Whilst there is a strong body of evidence demonstrating the importance of sleep to overall health and wellbeing, and literature devoted to treatment pathways for patients with insomnia within primary care [20, 21], studies have demonstrated that practitioners within this setting are often not assessing insomnia, or are uncertain regarding making the diagnosis [22]. Reasons for this may include factors associated with the primary care environment or physician and include: time pressure especially during initial consultations [23, 24], workload [25], and knowledge including potential uncertainty regarding the diagnoses of insomnia symptoms compared with other sleep disturbances [19, 26, 27], which may interact to impair professional decision-making [28].

Alternatively, patient-related factors may hamper accurate assessment of insomnia complaints. Indeed, patients with insomnia symptoms may not always volunteer information about sleep to their physician without prompting [18]. This may be compounded in chronic insomnia cases, or be masked by a comorbid disorder including depression, anxiety or chronic pain. For example, Bartlett and colleagues [6] estimated that in 75% of cases where patients indicated insomnia was a problem for > 12 months, only 11.1% had discussed their insomnia with their physician during the previous year. To date, there has been limited research examining barriers to seeking assistance for insomnia from the patient perspective.

Given the importance of identifying and managing insomnia, this paper examines barriers to the assessment and diagnosis of insomnia from both the clinician and patient perspective, applying the Theoretical Domains Framework, given that it provides a synthesising architecture, allowing for relevant summaries of behavioural barriers and facilitators to promote progress and facilitate shared understanding [29]. Such a frame is useful given that successful behavioural and public health interventions are based on an understanding of the contexts in which they occur [30], and unlike other frameworks, the TDF includes social and environmental factors as areas for examination of relevant barriers and facilitators.

### The theoretical domains framework (TDF)

The Theoretical Domains Framework (TDF) was developed following collaboration of behavioural and implementation scientists who identified theories relevant to informing behaviour change, and grouped these constructs into domains in order to identify influences on the behaviour of health professionals in the implementation of evidence-based recommendations. The TDF has been widely used to examine challenges to the implementation of best practice within health care settings, having been cited > 800 times [31]. For example, the TDF has been used to examine: factors hindering nurses and midwives from engaging with pregnant women about stopping smoking [32]; barriers to the implementation of good hand hygiene practices [33]; and retrospectively to understand barriers in family practice faced by both clinicians and patients. For example, Yamada et al. [34] identified barriers for physicians, allied health workers, and patients in their adherence to treatment for asthma. The present paper utilises the TDF in a qualitative, and retrospective manner, to examine barriers to assessing insomnia from both the clinician and patient perspective.

## Methods

### Literature search and study identification

Articles examining barriers to the detection of insomnia in family practice were reviewed, and grouped according to whether they represented ‘clinician’ or ‘patient’ factors. MEDLINE and Google Scholar databases were utilised to identify relevant articles, with no restriction placed on publication date. Articles were identified using combinations of the following key search terms: insomnia, family practice, general practice, and sleep disturbance. In addition to using keyword searches, the databases were used to identify papers that had cited relevant articles identified in keyword searches. Reference lists of identified articles were also examined to identify other relevant literature. Only articles reporting original data, and published in English were included. Articles that specifically considered barriers to the assessment and diagnosis of insomnia from either the clinician and/or patient were considered, rather than those reporting prevalence data only. Using this approach, 19 articles were identified for subsequent analysis.

A narrative rather than systematic approach was used in the present analysis given that we were interested in using the TDF to identify behavioural barriers reported in studies, rather than aggregating results of these previous studies systematically [35].

### Application of the TDF framework to identified studies

Following study identification, we used the TDF as a framework in order to provide a theoretical lens through which to examine affective, cognitive, environmental and social influences affecting the identification of insomnia from the clinician and client perspectives within family practice [36]. This was done using Version 2 of the framework, where the relative domains have undergone validation, and as such we used the definitions and component constructs provided [33].

Each study was reviewed as to the relevant domains of the TDF providing the barrier to the recognition and/or assessment of insomnia in family practice. These include: ‘knowledge’, ‘skills’, ‘social/professional role/identity’, ‘beliefs about capabilities’, ‘optimism’ ‘beliefs about consequences’, ‘reinforcement’, ‘intentions’, ‘goals’, ‘memory, attention and decision processes’, ‘environmental context and resources’, ‘social influences’, ‘emotion’, and ‘behavioural regulation’ [30, 33].

## Results

### Clinician factors

Multiple barriers impact on a physician’s ability to identify and assess insomnia (see Table 1). Applying the TDF to these barriers demonstrates that these most commonly fall on the following domains: Knowledge, Skill, and Environmental Context and Resources. These barriers include a lack of physician awareness of the negative physical and mental health impacts associated with insomnia, as well as limited training opportunities to develop expertise with sleep problems [17].

**Table 1:** Studies of general practitioners: measures and outcomes relating to insomnia

Authors (Year) [Ref]	Country (community/cohort)	Study population	Measures	Main findings	TDF Domain(s)
Orr et al. (1980) [37]	United States	378 Physicians attending a course on sleep disorders	Examination of popular misconceptions of sleep (20 Questions)	Physicians scored below chance suggesting a greater need for sleep medicine education as part of training.	Skills
Hohagen et al. (1993) [38]	Germany	2512 patients attending 10 general practitioners	Questionnaire at 3 time points: baseline (T1), 4 months later (T2), 2-years later (T3), included DSM criteria	In 8.8% of cases of mild insomnia, 21.9% of cases of moderate insomnia and 39.2% of cases of severe insomnia the GP was aware of a sleep problem. 5% of insomnia cases were diagnosed without the patient reporting a sleep problem in the questionnaire.	Knowledge, Skills
Hohagen et al. (1994) [39]	Germany	330 older adults (aged 65+) attending 5 general practitioner clinics	DSM-III-R criteria	In 18% of cases of mild insomnia, 31% of cases of moderate insomnia and in 52% of cases of severe insomnia the GP was aware of a sleep problem. 14% of insomnia cases were diagnosed without the patient reporting a sleep problem in the questionnaire.	Knowledge, Skills
Haponik et al. (1996) [40]	United States	20 experienced primary care practitioners, 23 uninstructed medical interns and 22 interns with instruction on sleep medicine	Frequency of sleep history recorded during encounters with simulated patients (30 min consultations)	Interns who had received instruction in sleep medicine more often asked about sleep (81.8%), but uninstructed interns (13%) and physicians (0%) did not record sleep history during consultation.	Knowledge, Belief about capability
Papp et al. (2002) [41]	United States (Northeast Ohio)	105 physicians	Structured survey on attitudes and knowledge of sleep disorders	Physicians rated their knowledge of sleep disorders as ‘fair’ (60%) and ‘poor’ (30%). Only 10% rated their knowledge as good, and 0% rated it as excellent.	Knowledge, Skills, Professional Role and Identity,
				Greatest influence on changing practice style regarding sleep were journal articles followed by continuing education, followed by discussion with specialists.	
Siriwardena et al. (2010) [42]	United Kingdom (Lincolnshire, rural cohort)	Cross-sectional study of GPs (*n* = 84)	Prescribing preferences of GPs for insomnia vs anxiety diagnoses	For insomnia, GPs were more likely to favour giving advice on sleep hygiene and prescribing a hypnotic (Z-drugs favoured over benzodiazepines). For anxiety, referral to a psychologist/mental health worker was favoured.	Beliefs about capabilities, Environmental context and resources
				Preference to reduce use of drugs for insomnia but GPs felt insufficient resources or alternative management strategies were available	
Hassed et al. (2012) [22]	Australia, Melbourne (metropolitan sample)	15 General Practitioners	Focus groups (*n* = 7) and face-to-face interviews (n = 8). DSKQ	Scores from DSQK suggested gaps in knowledge related to defining the underlying cause and correct treatment options.	Knowledge, Skills, Environmental context and resources
				Behavioural intervention were viewed as preferable to prescribing medication.	
				Barriers to knowledge identified: limited training, lack of resources, patient expectation to receive a pill, consultation time constraint.	
Cheung et al. (2014) [43]	Australia, Sydney (metropolitan sample)	GPs (n = 8) Pharmacists (*n* = 14)	Semi-structured interview from a convenience sample. Data analysed using a framework analysis	Practitioners perceived an overreliance on pharmacotherapy and inadequate support to direct patients to alternate pathways.	Environmental context and resources
				Patients often have a reliance or expectation of a ‘quick fix’.	
Conroy & Ebben (2015) [44]	University of Michigan Hospitals and Weill Cornell Medical College of Cornell University.	Physicians (*n* = 239)	Questionnaire –mailed out	Most physicians did not nominate CBTi or a hypnotic as the most effective treatment for insomnia.	Knowledge, Skills
				1/3 recommended sleep hygiene.	
				*N* = 22 felt CBTi alone was effective.	
Davy et al., (2015) [45]	Primary care in Nottinghamshire and Lincolnshire.	Health professionals (n = 23), and patients with insomnia (*n* = 28)	Focus groups, and interviews	Practitioners tended to focus on sleep hygiene rather than CBTi.	Knowledge, Skills, Behavioural Regulation
				Some practitioners felt they colluded with patients when prescribing hypnotics.	
				Patients often ignored sleep hygiene advice, and sometimes took hypnotics as not intended	
				Both practitioners and patients wanted more options and better training	

DSKQ = Dartmouth Sleep Knowledge Questionnaire; GP = General Practitioner (equivalent to family practitioner in USA)

Many family practitioners have described their knowledge of sleep disorders and treatment options as ‘fair’ to ‘poor’, particularly in the areas of sleep physiology and sleep architecture [22]. Whilst being able to detect that a person was having problems with their sleep, physicians reported difficulty in defining the underlying cause and/or identifying the correct treatment [22].

### Patient factors

Research considering insomnia diagnoses within family practice clinics have primarily considered the proportion of patients suffering from insomnia and likely co-morbid risk factors; patient’s reasons for seeking treatment; and their utilisation of particular treatments (see Table 2).

**Table 2:** Studies of patients: measures and outcomes relating to insomnia

Authors (Year)	Country (town and community)	Study population	Measures	Main findings	TDF Domain(s)
Kushida et al. (2000) [18]	United States (Idaho, rural cohort)	Primary care patients seen at the clinic over a 1 year period (1997–1998) *n* = 1249, all 18+. (participation rate 60.1% 1254/2087)	Questionnaires (focused on sleep disordered symptoms for insomnia, RLS, OSA), ESS, SF-36 – daytime functioning (face-to-face or mail-out/ Interviews	32.3% had insomnia (29.7% of men and 34.5% of women).	Knowledge, Skills
				14.1% experienced insomnia on a nightly basis.	
				State that patients have limited access to sleep specialists and a lack of training for physicians	
Aikens & Rouse (2005) [36]	United States (Urban population)	*N* = 700 consecutive attendees at primary care, screened for insomnia. 326 mailed a follow-up survey to which *n* = 180 responded	Questionnaires assessing insomnia, sleep quality, and daytime consequences of sleepiness and fatigue (ISI, PSQI, ESS, DBAS, MFIS)	Of the 180 responders, 72% had probable insomnia. Those who had discussed it with their physician (52% of those with probable insomnia) reported poorer overall health Those who were more educated, had >co-morbid symptoms, lower TST or > daytime dysfunction more likely to discuss	Knowledge, Behavioural regulation, Beliefs about consequences.
Morin et al. (2006) [4]	Canada, Quebec Province.	2001 French speaking adults aged 18+. Mean age 44.7	Telephone survey with insomnia defined as per the DSM-IV and the ICD-10	29.9% reported insomnia symptoms.	Behavioural regulation, Beliefs about consequences.
				13% had consulted a healthcare professional about their insomnia.	
				15% had used a herbal product, 11% a prescribed sleep medication, 3.84% an OTC drug and 4.1% alcohol to manage insomnia.	
				Daytime fatigue, psychological distress and physical discomfort were symptoms prompting individuals to seek treatment.	
Bartlett et al. (2008) [6]	Australia, New South Wales, (mixed urban-rural)	Postal survey of 10,000 people randomly selected from the electoral roll (5000 aged 18–24 and 5000 aged 25–64). 3300 responded. Direct contact with a random subset of non-responders (*n* = 100) was undertaken (response rate of 49%) by telephone.	Postal survey and direct contact. Survey included AIS and ESS.	Population weighted prevalence of insomnia = 33% and in 74.7% of these the complaint has been present for > 12 months.	Behavioural regulation, Beliefs about consequences.
				Population weighted prevalence of a visit to a doctor for insomnia = 11.1%	
				Risk factors for insomnia were: older age, daytime sleepiness, short sleep duration (< 6.5 h), reduced enthusiasm.	
				Self-medication for insomnia was common but often satisfaction with treatment was poor. For prescription drugs 39% of users were satisfied compared with 16% for OTC drugs and 25% for herbal products.	
Bailes et al. (2009) [27]	Canada (Montreal, city cohort)	*N* = 191 older patients (aged 50+) in primary care. *n* = 138 from 2 hospital-based sleep clinics (new referrals aged 18+).	Sleep Symptom Checklist- 21 items (insomnia, sleep disorders, daytime symptoms and psychological distress) they had discussed with their physician in the past year.	Primary care patients often have sleep symptoms they do not discuss, or discuss non-specifically.	Knowledge
			Subsequent PSG with primary care participants		
				Those referred to the sleep clinic were more likely to have discussed sleep problems (also younger and more males)	
				Those who completed PSG more likely to report sleep symptoms compared with those who completed questionnaire only.	
Dyas et al. (2010) [9]	UK (Lincolnshire, rural cohort	Patients (who had sought help for insomnia in the previous 6 months)	Focus groups/ semi-structured interviews separate for patients (*n* = 30, 11 M, 19 F, aged 25–70)	Patients felt a need to convince professionals of their health problems.	Beliefs about capabilities, Environmental Context and Resources
				Patients often suffered for long periods before seeking help, and had tried self-help methods	
				Patients recognised sleep problems were linked to detrimental outcomes.	
				Clinicians noted multiple causes of sleep problems	
				Clinicians often focused on underlying causes rather than addressing treatment or consequences of non-treatment.	
Omvik et al. (2010) [46]	Norway	Epidemiological postal survey (*n* = 5000). Mean age 48.1.	Sleep medication prevalence and reasons for use questions	Prevalence of sleep medication use: Lifetime = 18.8%, Current = 7.9% and Chronic = 4.2%.	Social influences
			Bergen Insomnia Scale, Global Sleep Assessment Questionnaire, Structured Clinical Interview for DSM., WHOqoL, SDS	Sleep medication use associated with low SES, older age, female gender, frequent sleep and/or mood disturbance.	
				Among those who had ever used a sleep medication, 80.3% would prefer a non-drug treatment.	
Senthilvel et al. (2011) [19]	United States (Cleveland Ohio, urban population)	New adult patients aged 18–65 (*n* = 101) 52% female, mean age = 38 years	CSHQ, Berlin, ESS, STOP, review of GP records of the consultation	30% of cases = possible insomnia, but limited screening and sleep history obtained during the consult	Environmental Context and resources
Bjorvatn et al. (2017) [15]	Norway	Patients visiting their GP (*n* = 1346), 35.9% Male	BIS, Self-reported sleep problems (1-item), insomnia (DSM-IV criteria), hypnotic use	BIS insomnia rate = 53.6%, sleep problems (single item) = 55.8%.	Knowledge, Skills
				Hypnotics used by 16.2% (daily use was 5.5%).	

RLS = Restless Legs Syndrome, OSA = Obstructive Sleep Apnoea, ESS = Epworth Sleepiness Scale, PSG = polysomnography, ISI = Insomnia Severity Index, Pittsburgh Sleep Quality Index, DBAS = Dysfunctional Beliefs About Sleep Scale, MFIS = Modified Fatigue Impact Scale, TST = Total Sleep Time, CSHQ = Cleveland Sleep Habits questionnaire, STOP = Rapid Screening Tool for OSA, AIS = Athens Insomnia Scale, WHOQoL = World Health Organization’s quality of life assessment. SDS = Severity of Dependence Scale., DSM- Diagnostic and Statistical Manual, ICD-10 = International Classification of Diseases, 10th edition. DSKQ = Dartmouth Sleep Knowledge Questionnaire, BIS=Bergen Insomnia Scale, GP = General Practitioner (equivalent to family practitioner in USA)

## Discussion

### Clinician factors

The present analysis has demonstrated that insomnia is encountered by those in family practice at a greater rate than that in the general population. This disparity likely represents general practitioners being the gatekeepers to further medical care [18]. Despite this role, there are substantial barriers to the recognition and diagnosis of insomnia. Using the TDF, we found from the clinician perspective that there are barriers related to: Knowledge or an awareness that insomnia is a significant issue in many cases, and/or a lack of training to identify insomnia as distinct from other sleep disorders. These barriers likely reflect a lack of education being included in training curriculum [9, 42, 44], and a lack of access to continuing education and professional development for primary care physicians [27, 47]. For example, Mindell and colleagues [47] reported that the average time spent on sleep tuition in medical school across countries was 2.5 h, with some countries (e.g. Malaysia, Indonesia, Vietnam) providing no formal education, and only three countries (Australia, Canada, and the United States) providing more than 3 h of education. The lack of education around sleep disorders can have dire clinical consequences. For example, Lu et al. [48] found that a third of patients attending a sleep clinic were suffering from obstructive sleep apnea (OSA), but had been prescribed a sedative to help them sleep, inadvertently increasing their risk of having a motor vehicle accident.

While education of physicians has been demonstrated to improve sleep knowledge [40], the present article has identified that many in family practice rate their knowledge as either ‘fair’ or ‘poor’ and that there are gaps in their knowledge when assessing insomnia [22, 41]. Summarising the core features of sleep that contribute to a diagnosis of insomnia provides a useful and informative resource for those in family practice [17] (see Table 3).

**Table 3:** Classification of insomnia: Important features^a^

Features	Classification	Clinical note(s)	Further classification or notes
Duration of symptoms			
	Acute/Short-term (ICD-3)	Symptoms last < 3 months	Typically lasts 1 night to a few weeks. May result from illness or a circadian rhythm disturbance such as jet-lag
	Chronic (ICD-3)/Insomnia disorder (DSM-5)	Symptoms last > 3 months	Usually trouble sleeping is reported 3+ nights for > 3 months
Timing			
	Onset	Falling asleep takes > 30 min	
	Maintenance	Interruptions lasting more than 30–45 min are experienced during the night	
	Early termination	Waking earlier than intended & unable to resume sleep	
Severity			
	Mild	Almost nightly complaint	little or no impairment on social or occupational functions
	Moderate	Nightly complaint	Mild-moderate impairment on social/occupational functions
	Severe	Nightly complaint	Severe impact on social/occupational functions

^a^Further information on these issues is provided by [1, 2, 49–52]

The present analysis demonstrates that the primary care environment itself makes it difficult for general practitioners to assess sleep. Clinicians are often ‘time poor’ during consultations, may be presented with a lengthy list of complaints, and/or be reluctant to address sleep complaints [18], especially if they are rated as less important than other symptoms [53]. For example, in the UK, it is estimated that only 50% of patients with insomnia seek help from their general practitioner, with patients often receiving only a sleep hygiene leaflet or a hypnotic [42], which may have limited efficacy in treating their insomnia compliant [54], or be ignored by patients [45].

The timing of any insomnia assessment by a physician is also important given that chronic insomnia is unlikely to resolve on its own [21]. For example, previous studies have examined whether physicians assess sleep history during an initial consultation [19]. However, this initial meeting contains a myriad of priorities including the development of rapport and trust, learning about the patient and establishing the seriousness of any sleep-related complaints, all within time constraints. Hence, it may be difficult for a family practitioner to establish what constitutes a normal or impaired quantity and quality of sleep in new patients [55]. To date, limited studies have examined how general practitioner’s diagnose sleep in their regular patients, which is important given the chronic nature of the disorder, and that the recommended treatment (e.g., CBTi) may require multiple consultations and/or referral to a sleep specialist [16, 21].

### Patient factors

From a patient perspective, different barriers were identified including the Beliefs about Consequences, in this case that insomnia symptoms are associated with poorer outcomes. However, an important finding of the present study is that people who typically seek help for insomnia do so because of other symptoms which reflect outcomes associated with insomnia, including impaired daytime functioning [36], psychological distress or physical discomfort [4] rather than insomnia itself. Indeed, Dyas et al. [9] reported that some patients felt that they had to convince a medical professional as to the seriousness of their insomnia-related symptoms, highlighting the barrier of Beliefs About Capability, which affects a patient’s empowerment, confidence and self-esteem [31].

One barrier for patients identified in this study was Behavioural Regulation, reflecting that insomnia was more likely to be discussed only when symptoms were more complex, and/or daytime functioning was already impaired. For example, Morin and colleagues [4] reported that daytime fatigue, discomfort and psychological distress were factors likely to prompt individuals to seek treatment. Despite this, some patients who had reported suffering for long periods from insomnia felt a need to convince their health-care professional of their trouble sleeping [9], reinforcing the lack of awareness of the detrimental effects of insomnia on mood and performance by those seeking treatment.

We also identified that there is a knowledge barrier from the patient perspective, which likely represents a lack of awareness within the general public about the consequences of insomnia, and the available options regarding treatment. Given this identified barrier, targeting insomnia within the primary care environment is important from the perspective of both the practitioner and the patient. From the patient’s viewpoint, those who sleep better experience less overall distress, and are more likely to play an active role in the management of their disease. From the practitioner’s viewpoint, they are often the first point of call for any health or medical complaint and are in a position to provide the relevant support and resources [6].

There is often a patient expectation or Social norm barrier to receive a panacea “sleeping pill” in cases where insomnia has been a long-term problem, and also a reluctance from patients who have received a hypnotic drug to trial non-pharmacological approaches [42], despite hypnotics not being recommended as first-line treatment for insomnia [56]. This is supported by Australian data indicating high rates of sedative medication prescriptions to treat insomnia [57]. In addition, a recent survey of those in family practice and community pharmacists reported that practitioners perceive an overreliance on pharmacotherapy amongst insomnia patients [43]. One reason for this may be that patients have often tried self-help or relaxation methods prior to consulting a general practitioner and have lived with the condition for long periods prior to seeking help [9].

### Overcoming identified barriers

In order to improve recognition and assessment of insomnia in family practice settings, a multifaceted approach addressing both clinician- and patient-factors is likely to be more effective than a single intervention [58, 59]. Given the barriers include the Environmental Context specifically, suggested targets for improvement may include: increased awareness within the environment/clinic setting itself (e.g., chart reminders for physicians and infographic resources on the importance of discussing insomnia symptoms for patients); the provision of professional development resources and/or journal articles for physicians [41], increased education and training of those in primary care [17], and/or opportunities for brief interventions given the time barrier faced by those in family practice. For example, while > 80% of patients who had used a sleep medication would prefer a non-pharmacological treatment, many were not offered an alternative [46]. However, both a simplified sleep restriction intervention (which forms one part of CBTi) [60], and a shortened 5-session brief CBTi trial were found to increase both sleep quality and decrease insomnia symptoms during a 6-month trial [61].

A further avenue to overcome these barriers may include online competency training in sleep medicine which has proven effective for medical students [62], and could provide an avenue for continued training or professional development of physicians as part of continued professional development [63]. Indeed, a current trial is investigating whether use of i-Sleep, a guided online intervention will be feasible and effective for treating insomnia in primary care [64].

### Limitations and future directions

The TDF has been used extensively in health-related research [31], with an identified strength being its usefulness to researchers and practitioners from many disciplines. However, a limitation acknowledged in guidelines for the application of the TDF include that no formal guidance exists on how to apply the TDF. However, in the present article we present a strong rationale based on both literature and clinical acumen that the prevalence of insomnia symptoms encountered in family practice is high, yet as a disorder it remains largely under-recognised, and that the TDF provides an opportunity to synthesise knowledge in this area, allowing for relevant summaries of reported barriers and facilitators to promote progress and facilitate shared understanding. Future studies could examine the value of using narrative reviews along with frameworks such as the TDF to identify potential barriers and develop strategies to overcome these. Whilst undertaking specific qualitative studies provides in-depth understanding of the potential barriers, where there is substantial body of existing work that describes such barriers and facilitators it is a more responsible use of research to interrogate this data first.

Additionally, the application of the TDF does not allow for ranking or grading of studies based on their strengths and limitations. Future studies in this area should examine whether similar barriers and facilitators to other sleep disorders exist within family practice, and whether other allied health professionals (e.g., psychologists, social workers) encounter the same or different barriers to the recognition of insomnia to examine the generalisability of our findings.

## Conclusion

Despite the high prevalence of insomnia symptoms encountered in family practice, as a disorder it remains largely under-recognised, underdiagnosed, and undertreated. The present article has identified barriers to the recognition of insomnia centred on the clinician and the patient using the TDF. By focussing on the diagnosis of insomnia, this article bridges a gap between the identification and management of insomnia within the family practice setting and provides a useful resource for clinicians.

## Data Availability

Data sharing is not applicable to this article as no datasets were generated or analysed during the current study.

## Abbreviations

GP: General Practitioner
OSA: Obstructive Sleep Apnea
TDF: Theoretical Domains Framework
